# Understanding US-based Clinician Participation in Global Pediatric Hematology-Oncology Work: A Mixed-Methods Study

**DOI:** 10.5334/aogh.5224

**Published:** 2026-06-12

**Authors:** Kendall Carpenter, Nitin Shrivastava, Anna C. Revette, Natasha Archer

**Affiliations:** 1 Boston Combined Residency Program, Boston, MA, USA; 2 Department of Pediatric Oncology, Dana-Farber Cancer Institute, Boston, MA, USA; 3 Division of Pediatric Hematology/Oncology, Boston Children’s Hospital, Boston, MA, USA; 4 Division of Population Sciences, Dana-Farber Cancer Institute, Boston, MA, USA

**Keywords:** oncology, global health, pediatrics, capacity building

## Abstract

*Background:* Collaborative partnerships between institutions in high-income countries (HICs) and institutions in low- and middle-income countries (LMICs) are an effective tool to build global pediatric hematology-oncology (PHO) capacity and reduce survival disparities. However, clinicians at academic centers may face barriers that limit participation in global health work. Understanding these barriers and facilitators is essential to inform targeted interventions that promote sustained and meaningful engagement.

*Objectives:* This mixed-methods study, using an exploratory sequential design, aimed to identify barriers and facilitators to participation in global PHO work among clinicians at a single academic center in order to inform strategies to enhance engagement in global health initiatives.

*Methods:* Participants included faculty, nurses, and trainees from the Dana-Farber/Boston Children’s Blood Disorders Center (BC/DF). Qualitative interviews and a REDCap survey were utilized to explore current engagement, barriers, and preferences for global PHO work. Data collection occurred from June 2023 to May 2024, with thematic analysis and descriptive statistics applied to interview transcripts and survey responses, respectively.

*Results:* Thirty-eight clinicians (response rate: 11%) completed the survey, with 16% having prior global PHO experience and 50% expressing interest. Barriers included funding constraints, lack of protected time, and family obligations. Qualitative interviews (*n* = 15) identified additional barriers at the personal, interpersonal, organizational, community, and policy levels. Organizational barriers, such as academic promotion criteria and institutional support, were most prominent.

*Conclusions:* Despite interest, clinicians face substantial barriers to engaging in global PHO work, predominantly at the organizational level. Solutions proposed include enhanced institutional support, program management, formal educational curricula, and improved funding guidance. These findings underscore the need for tailored strategies to foster effective bidirectional partnerships and enhance global PHO capacity. Future research should explore perspectives from LMIC-based clinicians to optimize partnership effectiveness and sustainability.

## Introduction

Advancements in childhood hematologic and oncologic disorders are one of the great success stories of modern medicine. However, while these advancements have paved the way for pediatric cancer survival rates in high-income countries (HICs) as high as 80%, low- and middle-income countries (LMICs) have been left behind, with survival rates around 20% [1]. Similar disparities exist in patients with non-malignant hematologic disorders as well, such as sickle cell disease (SCD), which is now largely a chronic disease in HICs with most children born with SCD living into adulthood. However, roughly 95% of children with SCD live in LMICs, where 50%–90% of these children die before they are 5 years old [2]. It is clear that despite continuous advancements in care helping children with cancer and blood disorders live longer and better lives in HICs, these advancements are not yet reaching those in most parts of the world.

Bidirectional partnerships between medical institutions in HICs and LMICs are increasingly recognized as one of the best tools to help increase pediatric hematology-oncology (PHO) capacity and influence policy in LMICs [3–8]. It is clear that interest and engagement in global health work have increased across all levels of pediatrics in recent years [9–11]. However, pediatric academic hematology-oncology clinicians have many unique competing priorities. In this mixed-methods study, we aimed to identify barriers and facilitators to participation in global PHO work for clinicians interested in engaging with this work. Understanding these barriers will allow for more targeted solutions to help increase PHO faculty engagement in global health, thereby increasing the number, depth, and effectiveness of bidirectional global health partnerships.

## Methods

### Participants/Recruitment

Participants were recruited from faculty, nurses, and trainees at a Dana-Farber/Boston Children’s Blood Disorders Center (BC/DF). Purposeful sampling was used for initial qualitative interviews to recruit individuals known to be engaging in global health work (per study team’s prior knowledge and snowball sampling) (*n* = 12). Subsequently, all faculty (*n* = 150), nursing (*n* = 186), and trainees (*n* = 17) were e-mailed a REDCap survey to further explore barriers/facilitators. An additional three qualitative interviewees were recruited from the survey, in order to explore barriers with those who are not actively involved in global hematology-oncology work but are interested in engaging with this work.

### Data collection

Semi-structured interview guide (Supplementary File 1) was iteratively developed via research team consensus based on the primary research objectives and reviewed by a qualitative research expert (AR). The interviews explored: (1) interviewees’ current and past global health work, (2) facilitators and barriers to participating in global health work, and (3) ideal preferences for a global health program. Interviews were conducted from June 2023 to May 2024 by a research personnel trained in qualitative interviewing (KC) via Zoom. Interviews were audiotaped and professionally transcribed. Quantitative data collection was performed via REDCap survey from February to March 2024. Survey content was informed by initial qualitative interviews per the exploratory sequential design [12] as well as prior literature [11] and explored respondents’ interest in participating in global health work, types of work they would like to pursue, and barriers they have encountered. Weekly reminders were sent during the collection period; no incentives for survey completion were provided.

### Analysis

Iterative analysis was performed during data analysis to ensure meaning saturation [13]. A collaborative approach to codebook development was used, developed through comprehensive review of the interview transcripts and recurrent research team meetings/discussions. Both deductive codes based on the interview guide/research questions were used as well as inductive codes based on novel concepts that emerged from transcript review. To improve interpretive consistency of the codebook, two members of the research team independently coded two transcripts, compared coding, and clarified any discrepancies within coding structure (Supplementary File 2). Dual coding here was used as a heuristic tool rather than an attempt to quantify inter-rater reliability [14]. Coding was supported with NVivo 11. Descriptive statistical analysis of survey data was supported by Stata/MP 18.0. COREQ best practices for qualitative research were adhered to throughout [15].

## Results

### Survey

**Figure 1:**
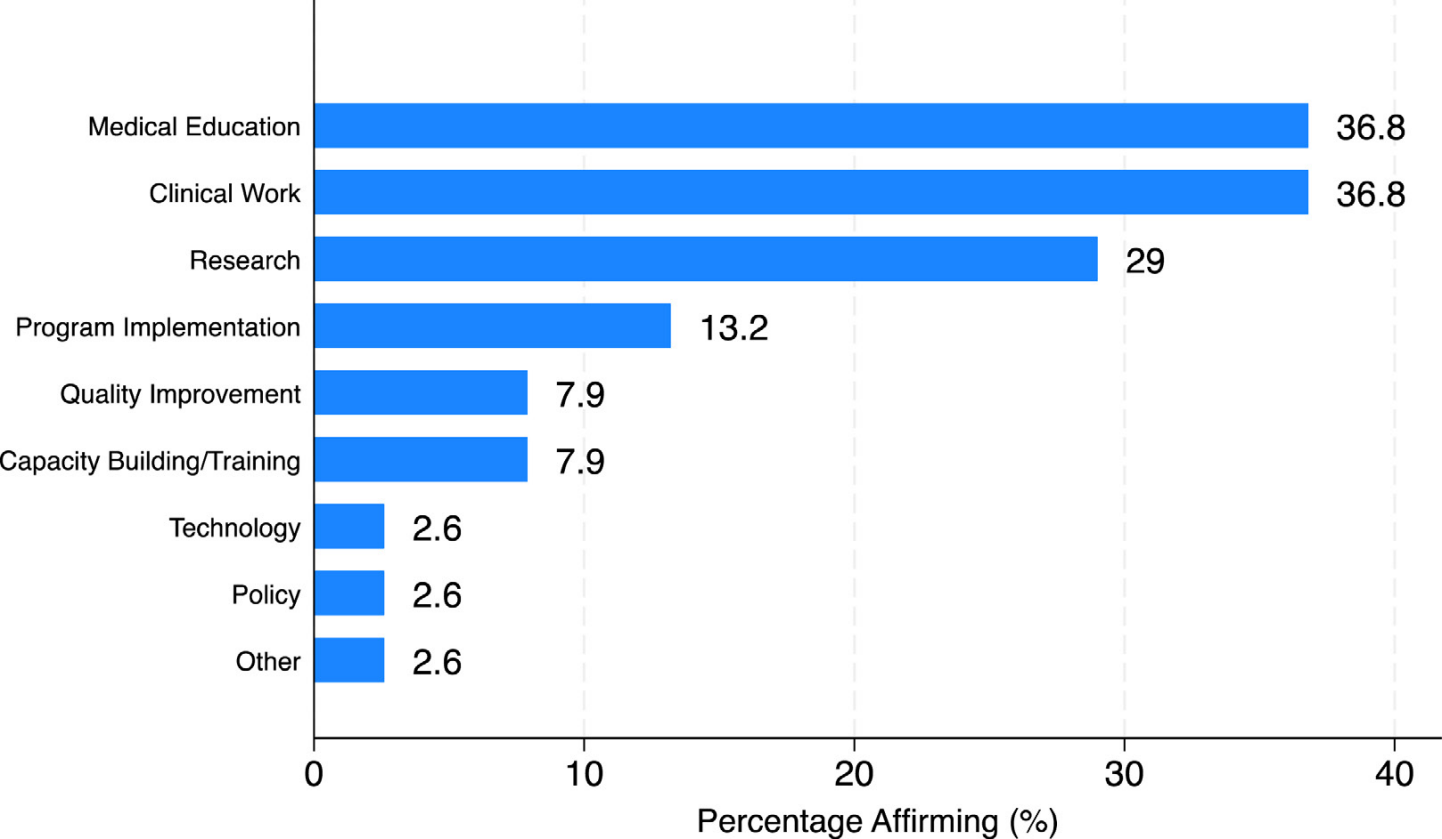
Interest in different global PHO activities, by percent affirming (*n* = 19).

**Figure 2:**
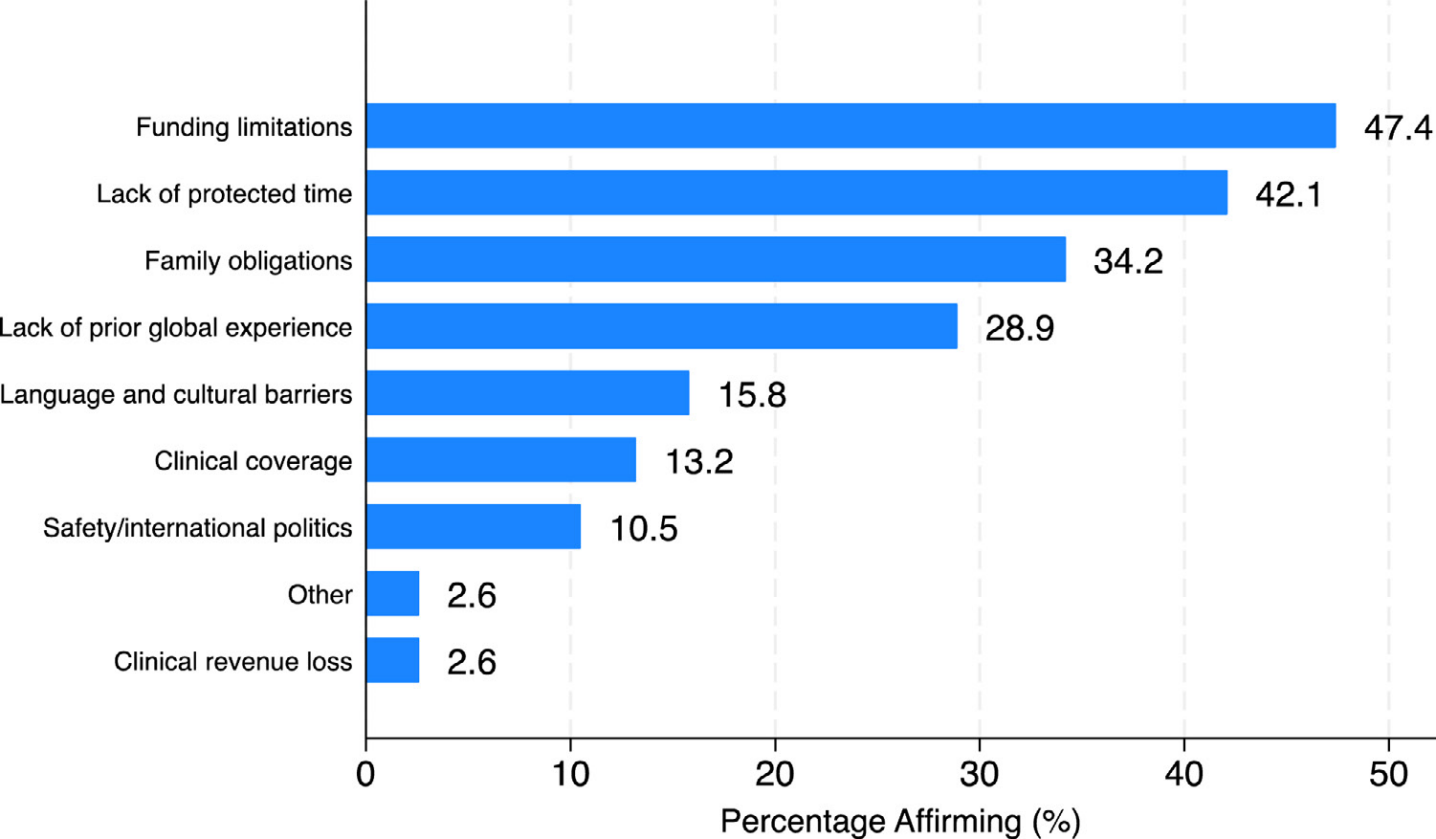
Barriers to global health participation, by percent affirming (*n* = 19).

A total of 38 clinicians completed the survey (11% response rate), with the majority of responses coming from attending physicians (*n* = 30, 78.9%) and the rest from fellows (*n* = 3, 7.9%), nurses(*n* = 3, 7.9%), and nurse practitioners (*n* = 2, 5.3%) (Table 1). The survey was not open to those who had already completed qualitative interviews. While only 6 respondents (16%) had prior experiences with global PHO work, 19 respondents (50%) expressed interest in pursuing global PHO work. Respondents were most interested in participating in medical education (*n* = 14, 56%), clinical care (*n* = 14, 56%), and research (*n* = 11, 44%) work (Figure 1). The key barriers identified to participating in global PHO work included funding limitations (*n* = 18, 72.0%), lack of protected time (*n* = 16, 64.0%), and family obligations (*n* = 13, 52.0%). Other less cited barriers include lack of prior global health work experience (*n* = 11, 44.0%), language or cultural barriers (*n* = 6, 24.0%), and clinical coverage (*n* = 5, 20.0%) (Figure 2).

**Table 1:** Baseline characteristics of survey respondents and interview participants.

INTERVIEW PARTICIPANT CHARACTERISTICS (*N* = 15)	*N* (%)
Gender identity Female Male	11 (73%) 4 (27%)
Type of sampling Purposeful Voluntary post-interview	12 (80%) 3 (20%)
Role Attending physician Trainee Nurse practitioner Nurse	9 (60%) 1 (7%) 2 (13%) 3 (20%)
**SURVEY RESPONDENTS (*N* = 38)**	***N* (%)**
Position Attending physician Trainee Nurse practitioner Nurse	30 (79%) 3 (8%) 3 (8%) 2 (5%)
Global health experience Yes No	6 (16%) 32 (84%)
Global health interest (*n* = 31) Yes No	19 (61%) 12 (39%)

### Qualitative interviews

Overall, 14 clinicians were approached for the initial interviews, and 12 consented to participate. After completing the survey, an additional three clinicians elected to participate in qualitative interviews (Table 1). Interviews lasted 15–40 minutes.

The vast majority of providers interviewed stated that they were doing less global health work currently than they would like to be doing ideally. One provider who is actively engaged in a global health fellowship program felt like they were doing the right amount of work. Another provider expounded that their global health work seems to come in waves, so at times she has felt like they are doing more than they would like to be doing, but that is often due to the competing priorities. One provider stated:

“*I think I’m doing less than I’d probably want to be doing, but at the same time I don’t know if I would have the time to do more, particularly the way that things are set up now and because I’m already involved in so much, you know, domestically…so even if somebody gave me the opportunity and said hey, here’s this global health opportunity, I don’t know if I could say yes right now because I just don’t really have the bandwidth*.” (Participant 6, Attending)

**Figure 3:**
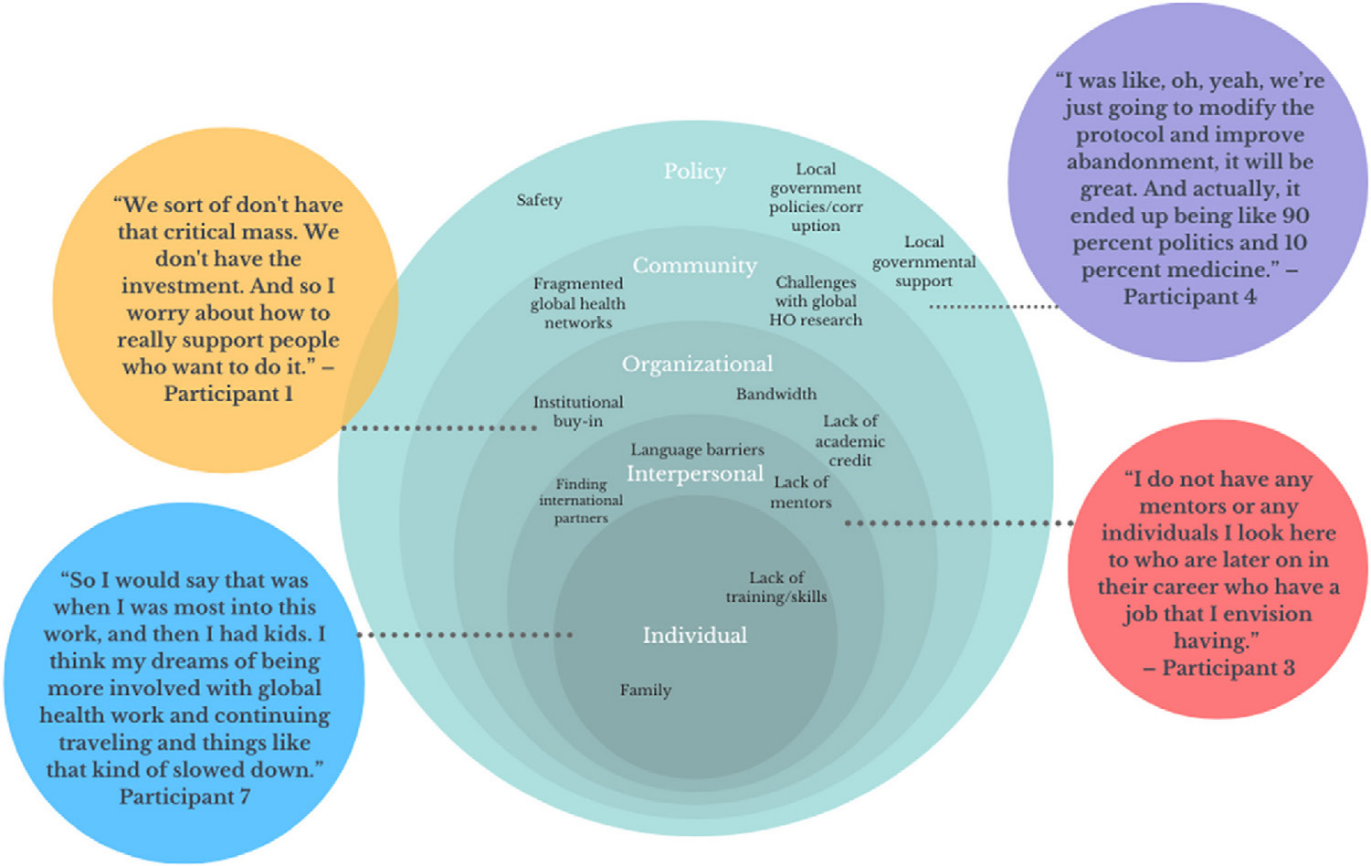
Barriers to participation in global PHO work mapped on the social-ecological model.

During transcript review, coding, and team meetings, the team identified concepts that aligned and echoed the social-ecological model, which was then used as the organizing structure for thematic analysis, demonstrating the complex interplay between individual, interpersonal, institutional, community, and policy-level barriers to PHO clinician participation in global health (Figure 3). The most frequent personal-level barrier mentioned was family commitments. While discussing family commitments, one clinician stated:

“*So I think it is difficult with a family to be away for the amount of time it takes to do global health…I mean, you really can’t do global health without being there, right? So you really – and really, to travel to the places we’re talking about, a week is a very short period of time. So it really requires being on site, I would say at least two weeks to understand what’s going on and to have some impact. And also, it needs to be more than once a year, so that’s a lot of travel*.” (Participant 1, Attending)

Other personal-level barriers mentioned were lack of individual’s skills or experience, making it difficult for them to do global PHO work well, or making it so they shied away from the work because they did not feel as if they were equipped.

Interpersonal-level barriers were also a major challenge to clinicians and included language barriers, lack of mentors, and difficulty identifying international partners. Many clinicians highlighted the benefit of speaking the language of the country you are working in, and some felt like discordant languages made it more difficult to engage in global PHO work in that region. Many felt that finding mentors doing global PHO work was difficult, with one clinician stating:

“*So I think that being an institution that does not specifically prioritize global health makes it a little bit more of an individualistic experience. I do not have any mentors or any individuals I look here to who are later on in their career who have a job that I envision having*.” (Participant 3, Trainee)

Others highlighted the difficulty in finding international collaborators with the bandwidth to engage in bidirectional partnerships given the demands of being a practicing PHO clinician, particularly in LMICs:

“*Part of it is just finding the right collaborators. Every time you do a multi-site study, you need to find people to work with who can carry a study out. And I think when you think about global oncologists, pediatric oncologists, they’re so overextended. They don’t typically have a lot of protected time for research*.” (Participant 1, Attending)

The most significant number of identified barriers (as well as potential solutions) were at the organizational level. Of note, all physicians interviewed (*n* = 10) mentioned challenges with academic promotion, whereas nursing colleagues focused more on bandwidth and competing demands. As one physician stated:

“*Outside of limited circumstances at different institutions, it is quite challenging to sustain an academic career with global health as a primary scholarly focus given that your career depends on you obtaining grants and funding to conduct both the work that you want to do, but also support your own salary for the time that you’re not clinically working*.” (Participant 3, Trainee)

Another significant barrier identified was the lack of institutional buy-in and feeling like global health work was not part of the institution’s strategic mission. A related but slightly distinct barrier was the lack of coordinated efforts within the institution.

Community-level barriers included fragmented external global health networks and the challenges with conducting global health research, both in terms of Institutional Review Board (IRB) approvals, implementation, and publishing. As one provider stated:

“*Obviously, the amount of need for global health work is enormous… but things get a little territorial…sometimes like different organizations will bump up against each other because they’re doing work in the same place, and I’ve noticed that it can cause some tensions. Which I feel like there’s so much to be done, like there shouldn’t be any limits on that.”* (Participant 4, Attending)

Policy-level barriers included some comments around safety, primarily as it pertains to travel during the COVID-19 pandemic. Many providers discussed how government policies, and sometimes the presence of corruption, impacted the work on the group, with on provider stating:

*“I think one thing I have learned from doing global health work…is that, if nursing programs at least are not adequately staffed, none of the rest of it matters. So if you think you’re showing up to teach IV skills, they don’t have time to sit there and learn or to really do anything, to come in and wanting to focus on – I don’t know – discussing signs and symptoms of whatever, F&N. There’s one nurse to 50 patients in [country]. So I think you cannot impact anything when stress around staffing is high.”* (Participant 7, NP)

Even beyond policy impacts, providers emphasized the importance of having local government support in your work early and how that can serve as either a huge barrier or facilitator depending on the existing relationship.

Providers were asked about potential solutions to these barriers with a focus on the organizational barriers. One of the most common suggestions was program management/lab manager support within the department in order to have a centralized coordinated global PHO effort. They paired this with desire for more regular meetings and programming for individuals engaged in this work and formal global PHO educational curriculum. As one provider stated:

“*Yeah. I think that we – I think that – I think there’s – my sense from talking to people over the years is a lot of people are interested in this type of work. And I think if there’s a way to pull resources or work together as a team to make this happen, I think that that would be the most helpful. And whether that’s a forum in which you can brainstorm ideas or practices that are going on and how can we all contribute? I think if, as a single person, it seems such a daunting challenge to establish this, but as a group with dividing – a divide-and-conquer mentality, that that might be more feasible. And whether it’s, I think, collaborating with other programs within Children’s would be helpful, too. But I think just having that forum of everyone that’s interested.*” (Participant 15, Attending)

While not explicitly stated, the global health nursing fellowship was identified as a significant facilitator for nursing involvement in global PHO work and thus an analogous track within the physician trainee course emerged as a potential solution. Beyond training, providers also wished for a formal academic track that recognized global health work. As one provider stated:

“*Maybe a slightly analogous thing is that I don’t think 20 years ago teaching paths were necessarily a thing in academic centers. And either you were producing high-end research papers and getting promoted, or you were a clinician, and now you can really grow your academic career through medical education. I mean, I could easily see that being created for global health.”* (Participant 4, Attending)

Finally, there was a call for both increased and new funding sources and formal guidance on how to access and apply for funding that supports global PHO work.

## Discussion

Pediatric hematologic and oncologic conditions remain a major contributor to the global disease burden, particularly in LMICs, where morbidity and mortality rates are significantly higher than in HICs [1,2]. Bidirectional partnerships aimed at increasing global PHO capacity have been recognized as a crucial tool for addressing these disparities [3–8]. However, our study reveals that while academic PHO clinicians at our institution are eager to engage in this work, most are currently doing less than they would like due to substantial barriers. These barriers to participation among US-based clinicians align with the social-ecological model, with organizational barriers identified as the most significant factor.

The social-ecological model, first introduced in the 1970s to explain how various systems influence individual health, has since been expanded to address human behavior [16,17]. During the thematic analysis of our study, we found that this model naturally mapped onto the barriers identified by clinicians, even though it was not part of the original study design (such as the interview guide or codebook). This finding suggests that multiple complex and interconnected systems influence clinician involvement in global PHO work, and all these factors must be considered when designing effective interventions.

Organizational-level factors were the most commonly identified barriers to clinician participation in global PHO work in this study. As a result, we specifically inquired about potential organizational solutions to address these barriers. The most frequently suggested solutions included support from program managers or lab managers, regular meetings or programming, formal educational curricula (including trainee pathways), the establishment of a global health academic track, and improved guidance on global health funding. Future work will prioritize these solutions based on feasibility and impact analysis. Recent survey research indicates a strong interest in global health training within PHO fellowships and supports many of the barriers identified in this study at the fellowship level [18]. Interest in global health within pediatrics has increased at both the trainee and faculty levels, indicating that the trend is not unique to PHO [9]. PHO programs can look to other pediatric specialties that have made further progress in global health support for guidance [19–21]. Additionally, a limited number of PHO institutions have developed advanced and mature global models, which can serve as examples for other institutions seeking to expand their global PHO efforts [22,23].

Our study should be considered within the context of its limitations. Originally conceived as an institutional needs assessment, it involves clinicians solely from a single urban academic center. While many barriers identified may be broadly applicable, some could be specific to our institution. Internally, this research will guide efforts to enhance provider engagement in global PHO work. The findings and proposed solutions could also aid other PHO departments seeking similar improvements, or conducting their own needs assessments under different circumstances. Additionally, effective bidirectional partnerships necessitate commitment from both sides. Further research is needed to explore barriers to participation from the perspective of PHO clinicians in LMICs, crucial for maximizing the impact of such partnerships [24]. Finally, this study was limited by the potential for volunteer bias as those who completed the study may be more inclined toward global health, particularly in light of the 11% survey response rate and modest number of total survey responses. Larger multi-site studies with broader role representation would help confirm and extend these results. The qualitative interviews, on the other hand, were designed to explore opinions of those involved or interested in global health by design using purposeful sampling so risk of volunteer bias does not apply to the qualitative portion of the study.

## Conclusions

There is significant interest in global PHO work, but major barriers exist to clinician participation. Targeted programmatic changes are necessary to support this interest and allow clinicians to meaningfully engage in this work.

## Figures and Tables

**Table:** 

DFCI/BCH	Dana-Farber/Boston Children’s Cancer and Blood Disorders Center
PHO	Pediatric hematology-oncology
HICs	High-income countries
LMICs	Low- and middle-income countries

## Additional Files

The additional files for this article can be found as follows:

Supplementary File 1. Qual interview guide.

Supplementary File 2. Codebook.

This finding of multiple local introgression events has at least three key implications.
