# Supplementary material for: Understanding US-based Clinician Participation in Global Pediatric Hematology-Oncology Work: A Mixed-Methods Study
Source: Ann Glob Health. 2026 Jun 12;92(1):54. doi: 10.5334/aogh.5224 (PMC13262642; doi:10.5334/aogh.5224)
Supplement: Supplementary File 1. — Qual interview guide. [file agh-92-1-5224-s1.pdf]

Barriers and Facilitators to Global Health Work
DFCI-BCH Global Health Task Force

Survey Provider Interview Guide

We will talk for about 20-30 minutes today, and as discussed earlier I will be recording our conversation. We keep everything confidential and anonymous and there will be no names attached to any write-ups or reports.

Being part of this discussion is voluntary and there are no right or wrong answers. We simply want to hear more about your ideas, perspectives, and experiences. You can skip any questions that you do not wish to answer, and you can end the conversation at any time.

We know that managing the many competing priorities during a career in academic heme-onc is challenging. We are hoping to learn more about how global health fits into your career, if at all, and barriers/facilitators to participating in global health work. When I refer to global health, I specifically mean any work that places a priority on achieving equity in health for all people with a particular focus on resource-limited settings. This work could be through research, program development, education, or policy work. We are interested in hearing about any global health work you have done while working at an academic medical center, whether that be while at DFCI or another institution.

To be respectful of your time and the task at hand, I may need to interrupt at times to move us on to different topics. Thank you for your cooperation if those circumstances arise.
Do you have any questions for me before we begin? [begin recording and confirm OK to audio record.]

I'd like to begin by hearing about your current role.

Have you previously been or are you currently involved in global HO work?

If yes:

- What motivated you to be involved in this work?
- How did you get involved with this work?
- How, if at all, has this work contributed or detracted from your career development?
- How did you support this work financially?

How would you describe your current level involvement in global health work?

- How do you feel about the amount of global health work that you are currently doing?
    - For example: Do you feel like you're currently doing **more** global health work than you'd like to be doing, **less** global health work than you'd like to be doing, or **the right amount** of global health work that you'd like to be doing?

What barriers, if any, have you experienced to participating in global health work?

- If they focus only personal life barriers, ask if there are any professional barriers they have encountered and if they cite only professional barriers, ask if there are any personal life barriers.
- Possible language: “In thinking about things outside professional realm, are there any other barrier, personal or otherwise, that you have noticed in participating in global health work"

We are interested in understanding what makes global HO work **meaningful** or **effective**.

- In your words what would make global health work **meaningful**?
- What resources/facilitators (for example mentors, partnerships, programs, funding sources, etc) do you think would be most important in making it a **meaningful** experience?

- In your words what would make global health work **effective**?
- What resources/facilitators (for example mentors, partnerships, programs, funding sources, etc) do you think would be most important in making it a **effective** experience?

If there is time and it has not been discussed earlier in the conversation:

- What resources or facilitators, if any, do you think would be helpful for our institution to provide to help facilitate global HO work (i.e. your global health supports wishlist)?

Any additional comment?

STOP RECORDING

Snowball sampling:
