# Supplementary material for: Understanding US-based Clinician Participation in Global Pediatric Hematology-Oncology Work: A Mixed-Methods Study
Source: Ann Glob Health. 2026 Jun 12;92(1):54. doi: 10.5334/aogh.5224 (PMC13262642; doi:10.5334/aogh.5224)
Supplement: Supplementary File 2. — Codebook. [file agh-92-1-5224-s2.pdf]

IRB-P00044519

Codes

Name	Description	Files	References
1. Current and past global health work		12	112
A. Location	Any utterances that describe/detail regions, countries, or cities where interviewee has previously or is currently working	12	31
B. Type of Work	Any utterances that describe/detail the type of global health work that the interviewee (including mentoring, activities related to global health including conferences, workshops, etc)	12	24
C. Motivations	Descriptions of initial and subsequent motivations	11	15
D. Career Development	If grants are talked about ways in which it aids their promotions/career development. Comments about improving their clinical expertise, resourcefulness, efficiency, etc	9	11
E. Personal Development	Happiness, satisfaction with life or work, etc	5	5
F. Financial Support	Where the money has come from? Support for interviewees time as well as the work done on the ground (in the form of salary travel, provided by specific grants, institution, or self-supported (interviewees own personal time). Doing work as part of current job (even if not in their job description).	12	16
G. Phase of career	Utterances describing at what point in career where they engaging with this work?	7	10
2. Perceptions on current level of engagement		12	17
A. Too much		2	2
B. Too little		10	13
C. Right amount		1	1

Name	Description	Files	References
D. Other		1	1
3. Barriers and Facilitators to Global Health Work		12	197
A. Funding	Both difficulty finding funding, but also sustaining funding (both grants, salary support). Discussion of "FTEs" as it relates to where the funding comes from.	11	21
B. Non-Work and Personal Life	Families/partners/children	10	14
C. Academic Advancement	Ability to get publications, publications with impact. Difficulty with specific reviewers. Promotion advancement issues including academic promotion tracts.	7	17
D. Other primary professional roles	Discussion of finding coverage, difficulty of balancing things outside of clinical work while on service, responsibility to primary patients, discussion of doing this work "on your own time," or not being the "main focus of your career." Lack of protected time during work hours to continue your work, vs. taking up weekend time/personal time. Discussion of "FTEs" as it relates to how they spend their time.	11	33
E. Personal safety	Safety as it relates to crime, COVID risks, or other health risks. COVID "putting things on hold."	7	9
F. Politics and Corruption	Feeling that the medical work is minimal without policy change/impact of corruption. Changes of political will impacting the work. Discussion of political structures outside of healthcare impacting the work.	4	11
G. In-country collaborators and partners	Any discussion of challenges or facilitators of finding and maintaining relationship with local collaborators and partners.	8	22
H. External global health networks	Discussion of external organizations (ie SIOP), hospitals (ie St Judes, etc) or institutions working in this space either as a support or barrier to interviewees global health work.	8	16
I. Coordination and community	Any commentary on lack of mentors/guidance, lack of global pediatric oncology community, lack or importance of leadership, commentary on individual work but not coordinated efforts. Individual effort. Lack of opportunities to get involved (particularly from the nursing perspective) or change in these opportunities over time. Idea of role specific opportunities.	11	26
J. Institutional buy-in	Not being part of the hospitals strategic mission.	6	14
K. Language, culture knowledge, and	Difficulty with communication. Lack of understanding of the culture/political environment of	5	7

Name	Description	Files	References
poor preparation for the work	the hospital. Being poorly prepared for the work due to these factors.		
L. Logistical cross-country research issues	IRB across sites/countries	3	7
4. Meaningful and effective work		12	44
A. Description or definition of meaningful work	Where and what they/their team did – describing the meaningful work. Why they feel it is meaningful (i.e. joy, can enact change, pride in work, interpersonal relationships made, etc)	11	11
B. Description or definition of effective work	Where and what they/their team did– describing the effective work. Why they feel it is effective (i.e academically productive, measurable change – improvement in survival rates, sustainability, infrastructure developed, sustainability, etc).	11	14
C. Description or definition of work without clear deliniation between meaningful and effective	Statements describing where and what they/their team did without clearly delineating between whether they are defining meaningful or effective work. Statements about meaningful work being the same as effective work.	2	2
D. Facilitators to meaningful work	Utterances explaining what helped make it meaningful work (i.e. ability to visit site multiple times, strong interpersonal relationships, etc)	5	6
E. Facilitators to effective work	Utterances explaining what facilitators contribute to effective work (i.e. clear vision/defined goals, institutional buy in, local champions, etc)	5	8
F. Facilitators to meaningfuleffective work combined		2	3
5. Wishlist for global health supports within our program	Hopes or ideas for ways to improve global health work within the DF/BC	9	11
6. Other	Any other utterances that do not fit in the above framework	12	61
